# Withaferin A combined with ricolinostat: a potent synergistic therapy for cervical cancer through regulating p53 ubiquitination and acetylation: WA combined with RIC regulates p53 ubiquitination and acetylation

**DOI:** 10.3724/abbs.2025048

**Published:** 2025-04-10

**Authors:** Tian Chen, Yiting Xu, Kunming Yang, Yutong Du, Zhuan Zhu, Lingling Xu, Xinrong Wang, Yi Yin, Yu Hu, Chengcheng Wang, Ronggui Hu, Chuanyin Li

**Affiliations:** 1 Medical College of Guizhou University Guiyang 550025 China; 2 Guizhou Mengxiang Qiancheng Technology Co. Ltd. (Science and Technology Park West Campus of Guizhou University) Guiyang 550025 China; 3 Department of Colorectal Surgery and Oncology (Key Laboratory of Cancer Prevention and Intervention China National Ministry of Education Key Laboratory of Molecular Biology in Medical Sciences Zhejiang Province China) the Second Affiliated Hospital Zhejiang University School of Medicine Hangzhou 310009 China

As a classic tumor suppressor gene, *p53* has been extensively studied since its discovery in the mid-1980s. Research findings have revealed that p53 protein expression is suppressed in various cancers [1]. For example, in cervical cancer, *p53* predominantly exists in a wild-type form to maintain its biological function [2]. Nevertheless, its tumor-suppressive activity is significantly impaired because of rapid protein degradation, short half-life, and low levels. Post-translational modifications (PTMs) of p53, such as ubiquitination, acetylation, phosphorylation and methylation, are critical regulators of its stability, activity, conformation, localization, and interactions with cofactors [3]. Among these, ubiquitination and acetylation play central roles in controlling p53 protein stability and activity [4]. Therefore, targeting p53 PTMs to modulate its ubiquitination and acetylation levels represents an effective strategy to increase its stability and tumor-suppressive function, offering a promising avenue for cervical cancer drug development. In 99% of cervical cancers (high-risk human papillomavirus-positive), E3 ubiquitin ligase E6-associated protein (E6AP) mediates the ubiquitination degradation of p53 [5], whereas histone deacetylase 6 (HDAC6) deacetylates p53. In this study, we explored the possibility of combining the natural product withferin A (WA) with the HDAC6 inhibitor ricolinostat (RIC) to treat cervical cancer cells, with a focus on the ubiquitination and acetylation of p53 and the consequences for its stability. These results suggested that the combination of WA and RIC is more effective than either treatment alone in inhibiting the degradation and increasing the stability of p53, thereby synergistically slowing the onset and progression of cervical cancer. 

To assess the effects of the effective doses of WA and RIC on cell viability, cervical cancer cells, including HeLa, SiHa and Caski cells, were incubated with different concentrations of WA ( Figure 1A) and RIC ( Figure 1B) for 24 h. After incubation, cell viability was measured by CCK-8 assay. More materials and methods are shown in the Supplementary Materials and Methods. The results indicated that both WA and RIC significantly inhibited cell proliferation in a dose-dependent manner and significantly decreased cell viability. To evaluate the combined effects of WA and RIC in cervical cancer cells, CCK-8 assays were performed on HeLa, SiHa, and Caski cells. The results showed that the combination treatment reduced cell viability more than either drug alone did, indicating a potential synergistic effect. CompuSyn analysis revealed a synergistic interaction with a CI value of less than 1 ( Figure 1C). In addition, colony formation assay confirmed that the combination significantly decreased the clonogenic survival of the cells ( Figure 1D). These results indicate that the combination of WA and RIC has a potent anti-proliferative effect on cervical cancer cells, providing an experimental basis for further research and application. 

Moreover, to investigate the effects of drugs on the growth of cervical cancer cells *in vivo*, HeLa cells were established and injected into BALB/c nude mice to establish xenograft tumors, which were allowed to grow to 80 mm³. After the mice were treated with the combination of 3 mg/kg WA, 10 mg/kg RIC, 3 mg/kg WA or 10 mg/kg RIC once a day for 14 days, the group treated with the combination of WA and RIC presented a significantly lower tumor volume than the control or other treated groups did ( Figure 1E). Moreover, the results revealed that tumors in the combination drug group weighed less than those in the control and single drug treatment groups did, indicating that the combination of the two drugs effectively inhibited tumor growth ( Figure 1F,G). However, no significant reduction in body weight was observed in the drug-treated groups, indicating that the combination therapy did not cause significant systemic toxicity, suggesting its potential safety for therapeutic use ( Figure 1 H). 

Taken together, these results suggest that the combination of WA and RIC has anti-proliferative effects on cervical cancer cells, although the exact mechanism of inhibition is unclear. The tumor suppressive capacity of p53 in most cervical cancers is compromised by its accelerated protein degradation, shortened half-life and reduced expression [6]. We therefore focused on p53 and investigated the effects of WA and RIC on p53 status. To investigate the effect of WA on the expression of p53 in cervical cells, we performed western blot analysis. WA treatment significantly increased p53 levels compared with those in the control group ( Figure 2A). To evaluate the stability of p53 after WA treatment, a cycloheximide (CHX) chase assay was used to monitor changes in the half-life of p53. The results revealed an increased half-life of p53 in WA-treated cervical cancer cells ( Figure 2B).

**Figure 1:**
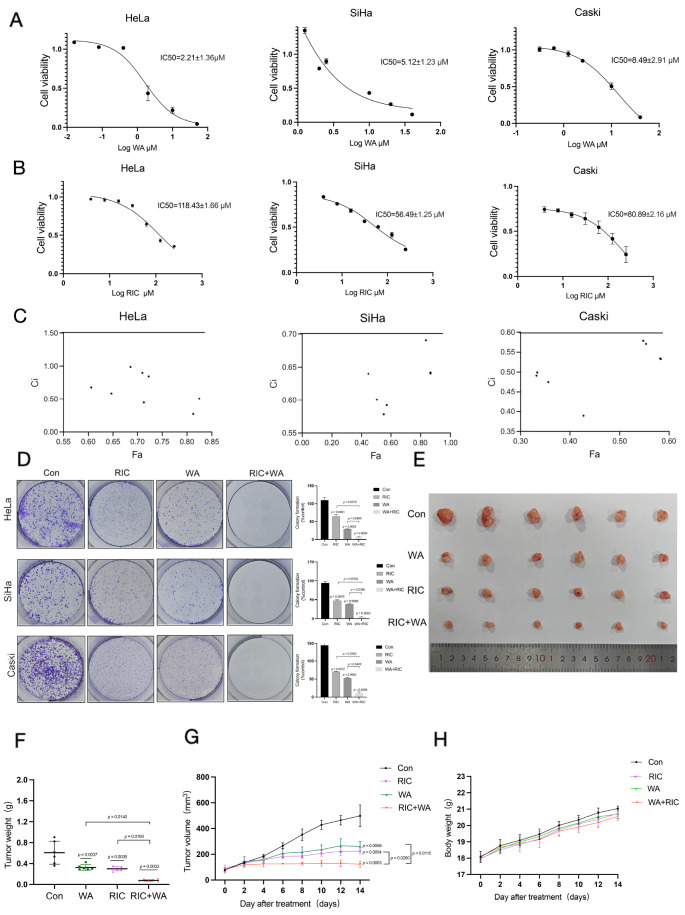
Figure 1 The RNA-binding domains (RBDs) of RNA-binding proteins (RBPs) play crucial roles in modulating various cellular processes implicated in carcinogenesis Each RBD is illustrated using distinct colors, and the sizes are accurately represented in terms of amino acid residues. Notable RBDs include the RNA-recognition motif (RRM), human heterogeneous nuclear ribonucleoprotein K-homology (KH) domain, cold shock domain (CSD), double-stranded RNA-binding domain (dsRBD), zinc-finger domain (ZnF), and YT521-B homology (YTH) domain.

**Figure 2:**
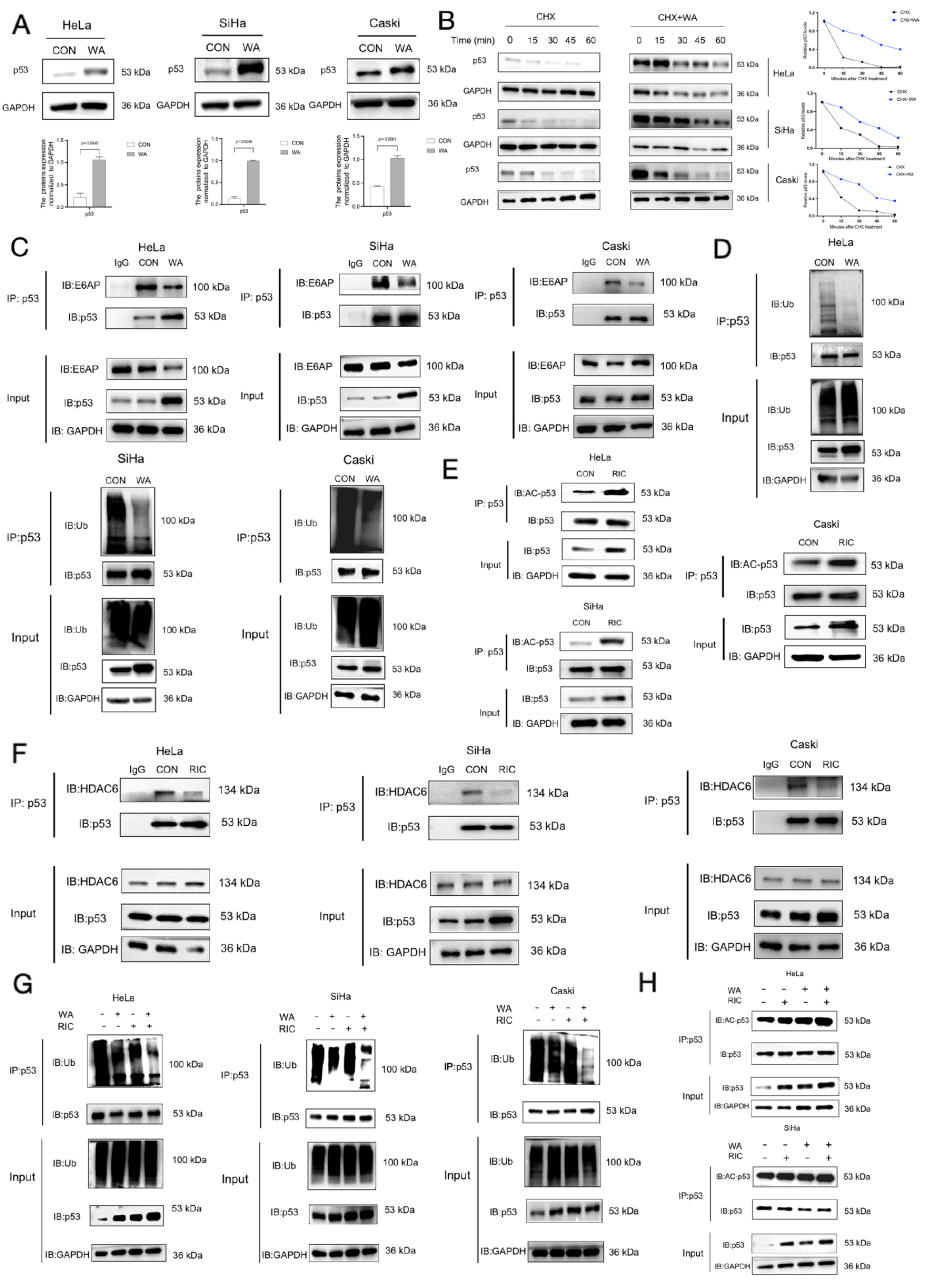
Figure 2 The combination of WA and RIC modulates the ubiquitination and acetylation of p53 in cervical cancer cells (A) Western blot analysis of p53 expression in cervical cancer cells treated with WA for 24 h. (B) Western blot analysis of p53 protein levels in cervical cancer cells first treated with or without WA for 24 h, followed by CHX treatment for 0, 15, 30, 45, and 60 min. (C) Co-IP analysis of the p53-E6AP interaction in WA-treated cervical cancer cells followed by western blot analysis. (D) Western blot analysis of p53 ubiquitination in cervical cancer cells treated with WA for 24 h. (E) Western blot analysis of p53 acetylation in cervical cancer cells treated with RIC for 24 h. (F) Co-IP analysis of the p53-HDAC6 interaction in RIC-treated cervical cancer cells followed by western blot analysis. (G) Western blot analysis of p53 acetylation in cervical cancer cells treated with WA, RIC, or both for 24 h. (H) Western blot analysis of p53 ubiquitination in cervical cancer cells treated with WA, RIC, or both for 24 h. All the experiments were performed in triplicate. Data are expressed as the mean ± SEM (n = 3).

Ubiquitination is a signal for protein degradation via the proteasome pathway [7]. Degradation of p53, which has been reported to be modulated by E6AP, an E3 ubiquitin ligase, is critical for the regulation of p53 levels in HPV-positive cervical cancer [8]. Hence, we performed co-immunoprecipitation (co-IP) and ubiquitination assays to elucidate the molecular interactions involved. WA treatment inhibited p53 binding to E6APs ( Figure 2C) and decreased p53 ubiquitination ( Figure 2D). This observation was independently validated in the HEK-293T exogenous expression system ( Supplementary Figure S1A). These findings suggested that WA may be a potential E6AP inhibitor. 

E6AP has been extensively studied for its role in mediating the ubiquitination and degradation of p53 [8]. Recent findings have revealed that HDAC6 regulates the level of p53 acetylation by deacetylating it, which further affects the stability and anti-tumor activity of p53 [9]. Therefore, the present study focused on HDAC6 and investigated the effects of the HDAC6 inhibitor RIC on p53 acetylation and its tumor-suppressive activities. Our results demonstrated that RIC treatment significantly increased p53 acetylation ( Figure 2E) and reduced the p53-HDAC6 interaction ( Figure 2F), as confirmed in the HEK-293T system ( Supplementary Figure S1B). To further investigate the combined effects of WA and RIC on p53 ubiquitination and acetylation, we treated cervical cell lines with both drugs and performed Co-IP assays. Compared with the control and single drug treatments, the combination of WA and RIC significantly increased p53 acetylation ( Figure 2G) and reduced p53 ubiquitination ( Figure 2H). 

This drug combination robustly suppresses the viability and proliferation of cervical cancer cells and inhibits tumor growth. This effect is achieved by effectively inhibiting p53 ubiquitination and promoting p53 acetylation, thereby stabilizing p53. Notably, RIC reduces p53 ubiquitination, whereas WA increases p53 acetylation. Interestingly, the level of p53 ubiquitination and acetylation changed more in the combined treatment group than in the single treatment groups. Considering the recent findings that E6AP promotes the deacetylation and degradation of HDAC6-mediated tumor suppressors [10], combined with the experimental findings, we hypothesize that there might be a cross-talk mechanism for the ubiquitination and acetylation of p53 in HPV-positive cervical cancer. However, further experimental validation is needed to confirm this hypothesis. 

In conclusion, this study establishes a promising therapeutic strategy for the clinical treatment of cervical cancer. In addition, our findings provide experimental evidence supporting the development of drug combinations that modulate the ubiquitination and acetylation of tumor suppressor factors in cancer therapy.

## Supporting information

25047Supplementary_materials-z

## Supplementary Data

Supplementary Data is available online at *Acta Biochimica et Biophysica Sinica* .
