# Supplementary material for: Withaferin A combined with ricolinostat: a potent synergistic therapy for cervical cancer through regulating p53 ubiquitination and acetylation: WA combined with RIC regulates p53 ubiquitination and acetylation
Source: Acta Biochim Biophys Sin (Shanghai). 2025 Apr 10;57(12):2133–6. doi: 10.3724/abbs.2025048 (PMC12747991; doi:10.3724/abbs.2025048)
Supplement: 25047Supplementary_materials-z [file 25047Supplementary_materials-z.docx]

**Supplementary Materials and Methods**

**Reagents and antibodies**

Stock solutions of withaferin A (WA) and ricolinostat (RIC) (Yuan Ye Biotechnology Co. Ltd., Shanghai, China) were prepared in DMSO (Sigma Aldrich, St Louis, USA). Dulbecco’s modified Eagle’s medium (DMEM), Opti-MEM, fetal bovine serum (FBS), penicillin/streptomycin (PS) and trypsin were obtained from Thermo Fisher Scientific (Waltham, USA). Monoclonal anti-GAPDH antibody, polyclonal anti-Flag tag antibody, polyclonal anti-Myc tag antibody, and monoclonal anti-His tag antibody were purchased from Proteintech Group, Inc. (Rosemont, USA). Antibodies against E6AP, p53, HDAC6, and ubiquitin (Ub) and normal mouse IgG were purchased from Santa Cruz Biotechnology, Inc. (Santa Cruz, USA). Goat anti-rabbit IgG-HRP and goat anti-mouse IgG-HRP were purchased from Jackson ImmunoResearch, Inc. (West Grove, USA). Protein G agarose was purchased from EMD Millipore Corporation (Burlington, USA). Cell Counting Kit-8 (CCK-8) was purchased from Beyotime Biotechnology (Shanghai, China). Cycloheximide (CHX) was purchased from Selleckchem (Houston, USA).

**Cell culture and transfection**

The human embryonic kidney cell line 293T (HEK293T) and the cervical cancer cell lines HeLa (HPV18-positive), SiHa (HPV16-positive) and Caski (HPV16-positive) were obtained from the Cell Bank of the Chinese Academy of Science (Shanghai, China), cultured in DMEM (high glucose), supplemented with 10% FBS, 100 mg/mL streptomycin and 100 U/mL penicillin (Gibco, Carlsbad, USA), and placed in a humidified incubator with 5% CO_2_ at 37°C. The plasmids were transfected into the cells using Lipofectamine 2000 (Life Technologies, Carlsbad, USA) according to the manufacturer’s instructions.

**CCK-8 assay**

The viability of cervical cancer cell lines (HeLa, SiHa and Caski cells) was measured by the CCK-8 assay. Cells were seeded at a density of 5000 cells per well in 96-well plates and treated with varying doses of WA or RIC for 24 h. Afterward, 100 µL of CCK-8 was added to each well and incubated at 37°C for 1 h. The absorbance at 450 nm was detected using a microplate reader (Thermo Fisher Scientific). Cell viability was calculated via the following formula: cell viability (%) = (OD_treated_−OD_blank_)/(OD_control_−OD_blank_) × 100%.

**Colony formation assay**

Cervical cancer cell lines (HeLa, SiHa and Caski) were seeded into 6-well plates (1,000 cells/well). After 6 days of culture at 37°C, the cells were fixed with 4% paraformaldehyde (Beyotime Biotechnology) for 10 min at room temperature and then stained with 0.2% crystal violet (Beyotime Biotechnology) at room temperature for 10 min. Images were captured using an iPhone 11 camera (Apple, Inc., Cupertino, USA), and the number of colonies (≥ 50 cells) was manually counted via a light-field microscope (CKX53; Olympus, Tokyo, Japan).

**Western blot analysis**

The drug-treated cells were collected and lysed with RIPA buffer [50 mM Tris-HCl, 150 mM NaCl, 5 mM EDTA, 0.1% SDS (sodium dodecyl sulfate), and 1% NP-40, pH 7.5] supplemented with 1 mM PMSF at 4°C. Then, the protein samples were separated via SDS-polyacrylamide gel electrophoresis, transferred to PVDF membranes, blocked with 5% non-fat milk for 1 h, incubated with primary antibodies for 2 h, and then incubated with the corresponding secondary antibodies for 1 h at room temperature. Finally, the protein bands were visualized via an enhanced chemiluminescence system and analyzed using Image J software.

**Protein stability assay**

A protein stability assay was conducted using cycloheximide (CHX). Briefly, the cells were treated with 120 µg/mL CHX for 0, 15, 30, 45, or 60 min to inhibit protein synthesis. The level of p53 protein at each time point was detected by western blot analysis.

**Co-immunoprecipitation**

To examine the endogenous interaction between E6AP and p53, total protein lysates from cells were subjected to co-immunoprecipitation (Co-IP) using an anti-p53 antibody, followed by immunoblotting with an anti-E6AP antibody. Additionally, to investigate the exogenous interaction between HA-E6AP and Flag-p53, total protein lysates from transfected cells were incubated with anti-Flag beads at 4°C for 8 h. In parallel, the interaction between HDAC6 and p53 was assessed under similar conditions to explore potential regulatory mechanisms. For the endogenous assays, the cells were washed with PBS and lysed in Triton buffer containing 1% protease inhibitor cocktail at 4°C for 20 min. The cell lysates were then sonicated, and the mixture was centrifuged at 14,000 *g* for 15 min at 4°C. The antibodies were added to the supernatant, which was subsequently incubated overnight at 4°C with rotation. The products were subsequently added to agarose beads and incubated for 8 h at 4°C with rotation. The protein A/G beads were then centrifuged at 2000 *g* for 3 min and washed three times with Triton buffer. SDS loading buffer was added, and western blotting was performed to confirm protein interactions.

**Ubiquitination assay**

Briefly, cells were pre-treated with either individual compounds or a combination of compounds prior to lysis. After treatment, the cells were washed with PBS and lysed in RIPA buffer. The lysates were sonicated for 35 s and centrifuged at 4°C for 10 min to remove debris. Immunoprecipitation (IP) was performed with the corresponding antibodies, and the samples were incubated on a rotator at 4°C overnight. The pulled-down proteins were eluted by boiling in 2× SDS-PAGE sample buffer, and the ubiquitination levels were analyzed by western blot analysis using an anti-ubiquitin antibody. The eluted proteins were further validated by western blotting following IP with labelled antibodies.

**Acetylation assay**

The *in vivo* acetylation assay was conducted following established protocols with modifications. The cells were pre-treated with either individual compounds or a combination of compounds before lysis. After treatment, the cells were washed with PBS and lysed in RIPA buffer supplemented with protease inhibitors and deacetylase inhibitors to preserve acetylation. The lysates were sonicated for 35 s and centrifuged at 4°C for 10 min to remove debris. Immunoprecipitation (IP) was performed using an anti-p53 antibody, and the samples were incubated on a rotator at 4°C overnight. The immunoprecipitated proteins were eluted by boiling in 2× SDS-PAGE sample buffer, and acetylation levels were analyzed by western blot analysis with an anti-acetyl-lysine antibody. Protein loading was confirmed by re-probing the membrane with the corresponding antibodies.

**Nude mouse xenograft tumor model**

Five-week-old female BALB/c nude mice were used for the animal experiments. The mice were fed with a standard pellet diet and had free access to food and water. They were kept in a room with artificial lighting on a 12/12-h dark/light cycle. HeLa cells (7.5 × 10^6^ cells/mouse) were subcutaneously injected into the right backs of the mice. The mice were randomly assigned to four groups and administered with WA, RIC, combination or saline at doses of 3 and 10 mg/kg per day. The size of the heterografts was monitored every 3 days, and the volume was calculated using the formula: L × W^2^/2 (L: length, W: width). After 14 days, all the mice were euthanized to harvest xenografts.

**Statistical analysis**

A two-tailed Student’s *t* test or one-way analysis of variance (ANOVA) was performed using GraphPad Prism software version 8. Data are presented as the mean ± SEM calculated from at least three independent experiments. *P* < 0.05 was considered significantly different.

**Supplementary Figure S1A**


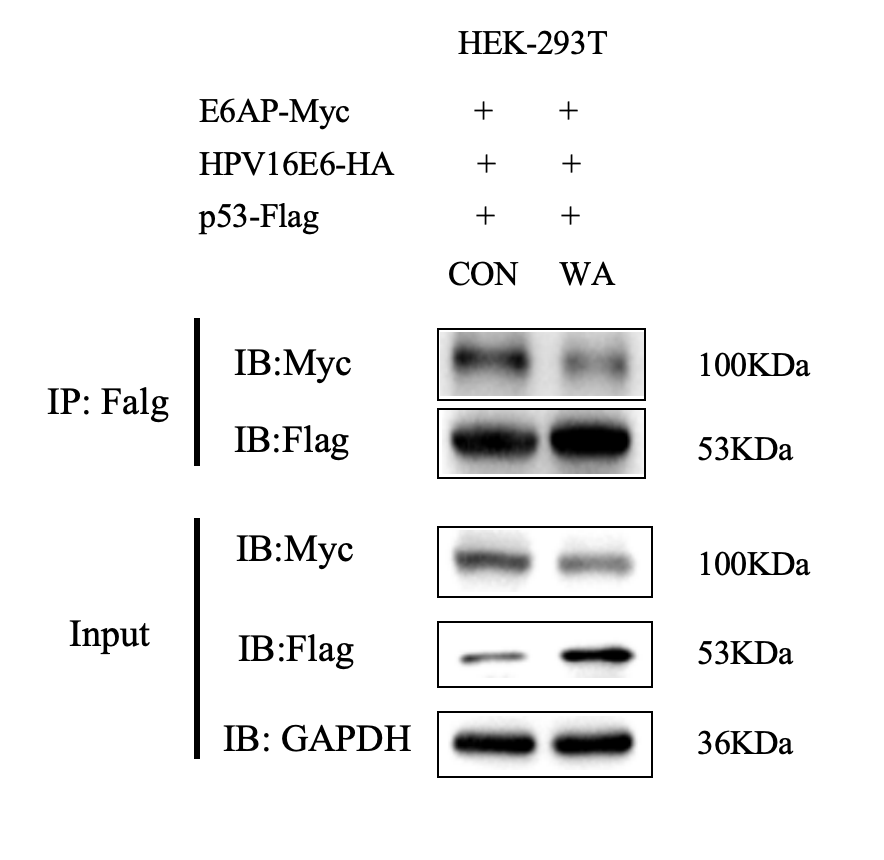


**Supplementary Figure S1B**


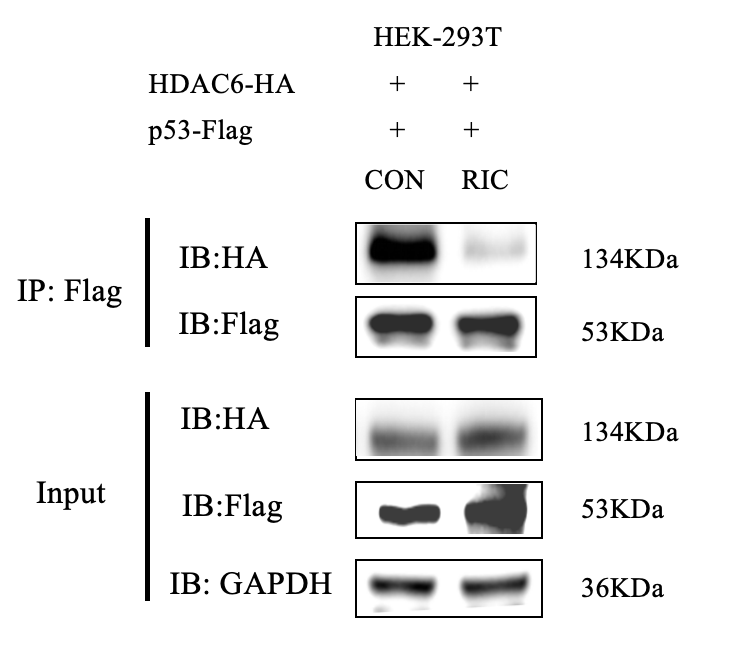


**Supplementary Figure S1. Withaferin A (WA) and ricolinostat (RIC) attenuate the binding of p53 to E6AP or HDAC6** (A) HEK-293T cells were transfected with E6AP-Myc, HA-HPV16 E6, or p53-flag. Co-immunoprecipitation experiments were performed using Flag beads. The cell lysates were analysed by western blot analysis. (B) HEK-293T cells were transfected with HDAC6-HA and p53-flag. Co-immunoprecipitation experiments were performed using Flag beads. The cell lysates were analysed by western blot analysis.
